# Impact of non-muscle cutting periumbilical transverse incision on the risk of incisional hernia as compared to midline incision during laparoscopic colon cancer surgery: a study protocol for a multi-centre randomised controlled trial

**DOI:** 10.1186/s13063-023-07162-x

**Published:** 2023-02-28

**Authors:** Soo Yeun Park, Gi Won Ha, Soo Young Lee, Chang Hyun Kim, Gyung Mo Son

**Affiliations:** 1 Colorectal Cancer Center, Kyungpook National University Chilgok Hospital, School of Medicine, Kyungpook National University, Daegu, Republic of Korea; 2 Research Institute of Clinical Medicine of Jeonbuk National University-Biomedical Research Institute of Jeonbuk National University Hospital, Jeonju, Jeonbuk Republic of Korea; 3 Department of Surgery, Chonnam National University Hwasun Hospital and Medical School, Hwasun, South Korea; 4 Department of Surgery, Pusan National University Yangsan Hospital, School of Medicine, Pusan National University, Yangsan, Republic of Korea

**Keywords:** Laparoscopes, Incisional hernia, Laparotomy, Wounds and injuries, RCT, Protocol

## Abstract

**Background:**

Minimally invasive surgery has become popular as a surgical approach for colorectal cancer because it has fewer complications related to the abdominal incision and perioperative complications. However, the incidence of incisional hernias in laparoscopic surgery has been reported to be similar to that in open surgery. We developed a new method, the non-muscle-cutting periumbilical transverse incision, for a small incision in laparoscopic colon cancer surgery. This study aims to evaluate the effectiveness of the non-muscle-cutting periumbilical transverse incision in comparison with the midline incision in reducing the incidence of an incisional hernia in patients undergoing laparoscopic colon cancer surgery.

**Methods:**

This is an open-label, multi-centre, parallel, superiority, and randomised trial. Altogether, 174 patients will be allocated in a 1:1 ratio to either the midline incision or the non-muscle-cutting periumbilical transverse incision group, after stratifying by the location of the tumour (right- or left-sided). The primary outcome of this study is the incidence of incisional hernias (both symptomatic and radiologic hernias) at 12 months after surgery. The secondary outcomes include operative outcomes, 30-day postoperative complications, pathological results, and patient-reported outcomes (short form-12 health survey questionnaire and body image questionnaire). Both primary (intention-to-treat) and secondary (as-treated principles) analyses will be performed for all outcomes. The statistical significance level was set at *p* < 0.05 (two-sided testing).

**Discussion:**

This trial may show that the non-muscle-cutting periumbilical transverse incision will reduce the incidence of incisional hernias compared to the midline incision.

**Trial registration:**

Clinical Research Information Service (CRiS) of Republic of Korea, KCT0006082. Registered on April 12, 2021.

## Background

Over the last three decades, clinical trials have shown that minimally invasive surgery is safe for patients with cancer. It has now become a popular surgical approach to treating colorectal cancer [1]. Minimally invasive surgery is less invasive than open surgery, which has a shorter postoperative recovery period and fewer complications related to a long abdominal incision. Most minimally invasive surgical procedures for colorectal cancer can be performed with a few 5–12 mm incisions for trocars, and a small incision is mostly used for specimen extraction. The length of the small incision is significantly shorter than that in open surgery. But it still has wound-related complications, and incisional hernias are one of the most common ones.

Incisional hernias deteriorate cosmesis and quality of life, increase the risk of bowel incarceration and reoperation, and result in additional medical costs. Even though the incidence of incisional hernias in laparoscopic surgery can be expected to be lower than that of open surgery, it has been reported to be similar to that in open surgery [2]. A literature review analysed 43 studies on laparoscopic colorectal resection and reported that the midline incision was the most commonly used approach for specimen extraction, followed by transverse and Pfannenstiel incisions [3]. The incidence of incisional hernia following the small incision was significantly higher following the midline incision than following the Pfannenstiel or transverse incisions. However, in prospective randomised controlled trials, the effectiveness of transverse incision in reducing the incidence of incisional hernia has not been proven [4]. There were no differences in pain scores or the incidence of incisional hernia between the two groups in a randomised controlled trial comparing the vertical periumbilical midline incision and the transverse left iliac fossa incision in laparoscopic anterior resection [5]. In another randomised controlled trial, a transverse incision was made outside of the linea semilunaris and rectus sheath. An intention-to-treat (ITT) analysis showed that the incidence of incisional hernia was similar in both groups [4], but the per-protocol analysis showed that it was significantly higher in the midline incision group. In this study, the failure of the primary outcome in the ITT analysis was because the protocol was violated in the transverse incision group. After all, the transverse incision is less versatile for either hand-assisted or open conversion.

Previous studies placed small transverse incisions for laparoscopic colon cancer surgery outside of the umbilicus, such as the iliac fossa incision or Pfannenstiel incision. We developed a non-muscle-cutting periumbilical transverse incision that is flexible to extract specimens of colorectal cancer and create extracorporeal ileocolic or colocolic anastomoses [6]. We hypothesised that this new transverse incision has benefits in terms of reducing the incidence of incisional hernia, as suggested by previous studies, while overcoming the cosmetic drawbacks of the standard transverse incision.

The current randomised controlled trial compares the incidence of incisional hernia between the midline and non-muscle-cutting periumbilical transverse incisions at 12 months after laparoscopic colon cancer surgery.

## Methods and analysis

### Study design

**Fig. 1:**
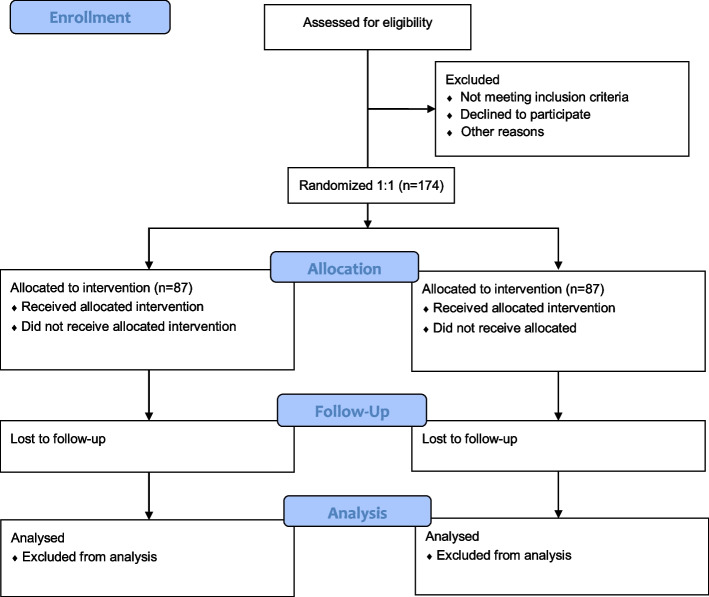
Flow diagram of the study protocol. SF-12, short form-12 health survey questionnaire

This multi-centre, open-label, parallel, superiority, randomised trial will compare the effectiveness of non-muscle-cutting periumbilical transverse incision versus midline incision on the incidence of incisional hernia in patients undergoing laparoscopic colon cancer surgery in the Republic of Korea at the following four tertiary hospitals: the Chonnam National University Hwasun Hospital, the Kyungpook National University Chilgok Hospital, the Jeonbuk National University Hospital, and the Pusan National University Yangsan Hospital. The study flow of the assessment, intervention, and follow-up is shown in Fig. 1.

### Participants

Eligible individuals are identified by the usual clinical team on a list of planned elective surgeries by reviewing patient information in the hospital’s electronic medical records at each institution. When eligibility is confirmed, surgeons will contact patients to provide details of the study background and process and request their participation in the study. Before the study protocol is initiated, each patient will be asked to provide written informed consent after the clinical investigator has explained the nature, significance, and scope of the clinical study appropriately and understandably (both orally and in writing). Clinical investigators are specifically trained medical doctors on the local study team. We began recruiting on April 20, 2021, and anticipate its conclusion in February 2024.

### Inclusion and exclusion criteria

Patients scheduled for elective laparoscopic colon cancer surgery at participating institutions are eligible if they meet the following inclusion criteria: (i) age ≥ 20 years; (ii) presence of pathologically confirmed colon cancer (adenocarcinoma, mucinous carcinoma, or signet ring cell carcinoma); and (iii) ability to understand verbal explanations, read instruction documents, and sign informed consent forms.

Patients meeting at least one of the following criteria are ineligible and were excluded from this trial: planned open surgery; expected incision length of < 1 or ≥ 10 cm; rectal cancer (lower border of tumour located within ≥ 15 cm from the anal verge); planned small incision outside of the umbilical area; palliative surgery for stage IV tumours; planned protective or permanent diversion; emergent surgery; current unhealed wound, fracture, peptic ulcer, or intraabdominal abscess; history of incisional hernia; and participation in any other interventional clinical trial within 6 months.

### Randomisation

Participant screening will be done by the surgeon and confirmed by the research coordinator of each institution. If potential participants meet all protocol eligibility protocol and agree to the informed consent forms, the investigators will enrol the participants and call the research coordinator of the initiating institution (Chonnam National University Hwasun Hospital) the day before the surgery and inform them of which group the registered patient is assigned to. In-hospital patients will be preoperatively allocated in a 1:1 ratio to either the midline or non-muscle-cutting periumbilical transverse incision groups, after stratifying by the location of the tumour (right-sided: caecum-transverse colon; left-sided: descending-rectosigmoid colon), according to the random sequence generated by a web-based randomisation system. The randomisation sequence will be generated independently by the designated staff not involved in outcome data collection and analysis before being provided to the randomisation contract for onward use. A unique study identification number will be assigned to the patients during data collection and analysis to ensure anonymityThe allocation information will also be blinded to the data analyst, and it will remain blinded to the public until a final comparison is performed.

### Blinding

The surgery team will not be blinded because the small incisions made during the surgery will be different in each case. Patients will also not be blinded, as the incisions will be visible during postoperative wound care. To reduce the bias associated with data collection, the outcome assessor will be separate from the surgery team and blinded to the allocation.

### Perioperative management

Patients in both groups receive treatment for colon cancer using the same strategy, according to the National Comprehensive Cancer Network Guidelines. Perioperative management is standardised at all institutions [7]. One day before surgery, all the patients will be completing the mechanical bowel preparation, except for those with right-sided colon cancer, for whom the preparation will be selectively administered according to the surgeon’s discretion. On the day of surgery, prophylactic broad-spectrum antibiotics will be administered before the incision. Surgery will be performed using a laparoscopic approach, for which one 11-mm camera port is placed at the periumbilical area and three or four 5-mm trocars are used. A modified complete mesocolic excision with central vascular ligation will be performed according to the location of the tumour [8]. A small incision for specimen extraction will be made according to group allocation. Stapled anastomosis will be performed extracorporeally through a small incision, except for cases of anterior resection, for which intracorporeal colorectal anastomosis is performed.

### Intervention

A small incision will be made by extending the periumbilical port for the camera scope in both groups. The size of the small incision will be determined based on the size of the tumour and the physical habits of the patient. The fascial closure methods are standardised as continuous closure using Stratafix (SF Symmetric PDS Plus®) with a 4:1 ratio (4-to-1 suture to wound length ratio) and bites of < 1 cm. The methods for closure of the subcutaneous fat and skin (skin stapler or 3–0 nylon vertical mattress) depend on the surgeon’s discretion. Patients randomly assigned to the midline group will undergo an incision along the midline skin, subcutaneous fat, and linea alba.

**Fig. 2:**
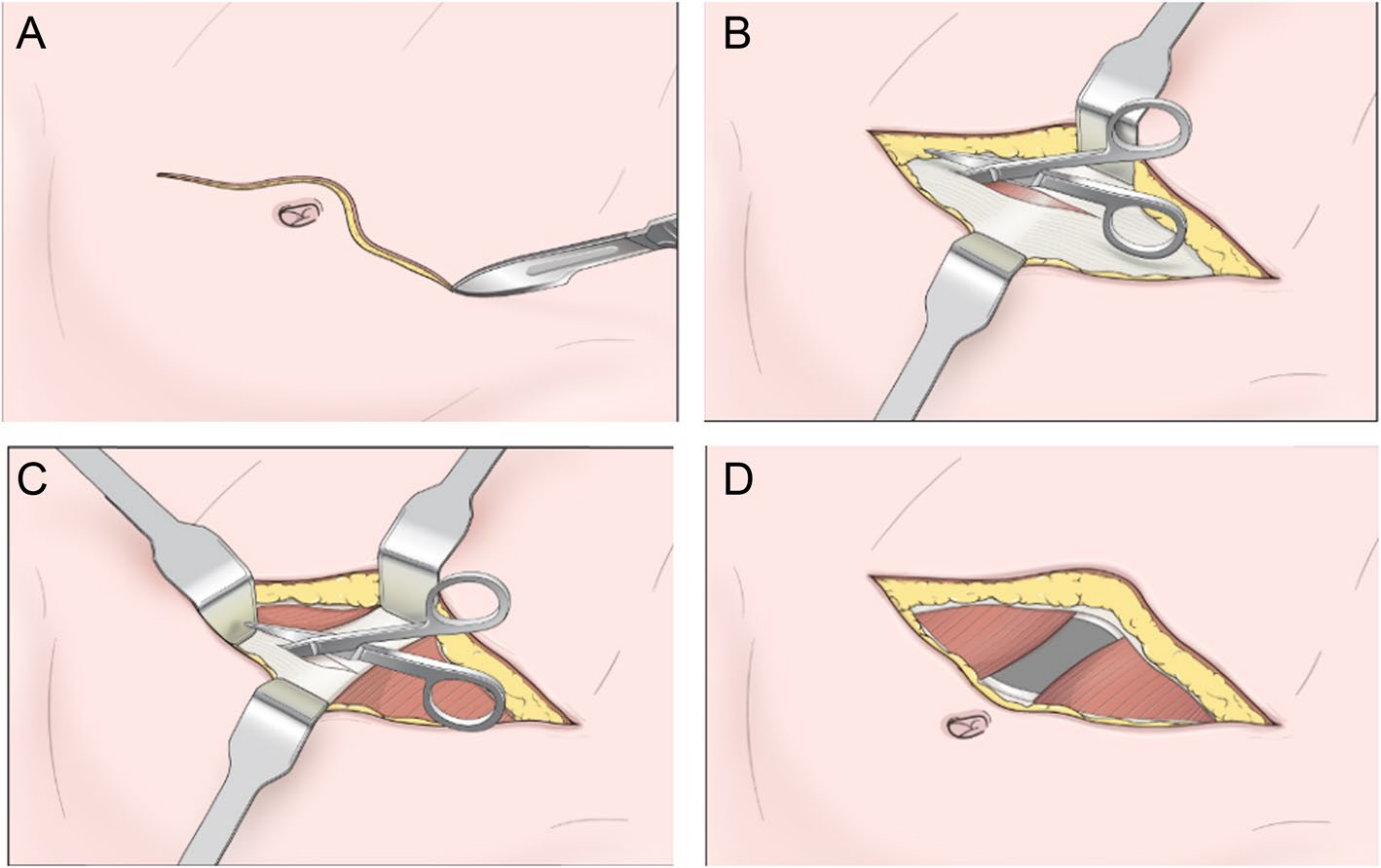
**A** Transverse skin incision. **B** Transverse incision of the anterior fascia of the rectus abdominis muscle. **C** Transverse incision of the posterior fascia of the rectus abdominis muscle. **D** Incision completed. Note. This figure was produced by Chang Hyun Kim in 2022. From **“**Periumbilical Transverse Incision for Reducing Incisional Hernia in Laparoscopic Colon Cancer Surgery,” by Chang Hyun Kim et al., 2022, World Journal of Surgery,46(4): p918. Copyright 2022 by SPRINGER

In the non-muscle-cutting periumbilical transverse group, the method of small incision is the same as in a previous report (Fig. 2) [6]. Briefly, the skin incision of the 11-mm periumbilical port will be extended transversely. Using monopolar electrocautery and crossing linea alba, the anterior and posterior rectus sheaths are transversely incised. With lateral traction of the rectus abdominis muscle with an army retractor, the posterior rectus sheath can be seen (Supplemental Video 1). The transversalis fascia and parietal peritoneum are further incised transversely. Continuous fascia closure will be separately performed for the anterior and posterior rectus sheaths. Implementing vertical or transverse incisions will not require alteration to usual care pathways (including the use of any medication), and these will continue for both trial arms.

### Outcomes (schedule of outcome measurement)

Data will be collected at the baseline (before surgery), during the hospital stay, at 30 days, and at 6, 12, and 36 months after surgery (Table 1). The primary outcome of this study is the incidence of incisional hernias, including both symptomatic and radiologic hernias, at 12 months after surgery. Incisional hernia is assessed by interviewing patients regarding subjective symptoms, performing a physical examination of the abdomen, and reviewing abdominal-pelvic computed tomography (CT) scans. An incisional hernia is defined as either a symptomatic hernia during the interview or a radiologic hernia when it is diagnosed on both an abdominal-pelvic CT scan and a physical examination in cases where subjective symptoms do not exist. The secondary outcomes are the length of hospital stay, estimated blood loss, pain numerical rating scale (0: no; 10: worst pain imaginable) score on postoperative days 1, 2, and 3; reoperation, open conversion, 30-day postoperative complications, (as classified by the Clavien–Dindo classification) [9], surgical site infection (as classified by the Center for Disease Control and Prevention criteria [10] and ASEPSIS score [11]), 30-day mortality, the incidence of repair of incisional hernias, pathologic result of colon cancer (pathologic stage, histologic type, differentiation, number of harvested and metastatic regional lymph nodes, lymphovascular, venous, and perineural invasion, and distant metastasis), and patient-reported outcomes (short form-12 health survey questionnaire before surgery, and at 12 months after surgery and Body Image Questionnaire at 12 months after surgery) [12, 13]. The incidence of incisional hernia at 36 months after surgery is another secondary outcome that will be separately analysed and reported after the last patient enrolled completes the follow-up.

**Table 1:** Schedule for assessment, interventions, and follow-up

Time point (visit number)	Baseline V0	Operation V1	1 month V2	6 months V3	12 months V4	36 months V5
Assessment						
Eligibility assessment						
Consent	X					
Demographics						
Baseline data	X					
Colonoscopy	*					
Operative outcomes		X				
30-day morbidity		X	X			
30-day mortality		X	X			
Symptomatic hernia			X	X	X	X
Radiologic hernia				X	X	X
Pathologic report			*			
Adjuvant chemotherapy				*		
Body image questionnaire					X	
SF-12	X				X	

Assessments undertaken as routine care for colon cancer are displayed with an asterisk

*SF-12*, short form-12 health survey questionnaire

### Withdrawal of patients

The participants are free to discontinue their participation at any time for any reason without any consequences. If a participant meets newly established or previously unrecognised exclusion criteria or has an urgent medical condition that disqualifies them from participating, the investigator may opt to remove them from the research. Patients who withdraw their consent after randomisation but before surgery will be replaced, whereas those who withdraw their consent after surgery will not be replaced. The data from these patients will not be included in the analysis.

### Data management and control

Participants’ identities will be kept private by using a research identification number that cannot be linked to their identities. Each centre will keep all patient-identifiable information in the file, apart from the data needed for analysis. To ensure consistent assessment, researchers will be uniformly trained. The result assessor at each centre will first input data into a registered paper-based case report form before entering it into the predesigned electronic version of the case report form. Both the paper and electronic versions of the case report forms will be maintained in a safe location, with only the members of the research team having access to them. All records will be stored for inspection at any time during and for 3 years after the completion of the study report. The study will be monitored by a committee of the Chonnam National University Hwasun Hospital.

Day-to-day support will be provided by the principal investigator (like supervision of the trial, recruitment, and medical responsibility of the patients) and the research coordinator (including data collection and follow-up of the patients) of each participating institution. The principal investigator and research coordinator will meet weekly. The Trial Steering Committee (TSC) will consist of representatives from participating institutions, which will act as a decision-making committee and be responsible for the scientific conduct of the study. The TSC will meet every 3 months, ensure that the trial is conducted following relevant principles, and provide overall supervision.

#### Frequency and procedures for auditing trial conduct

The Institutional Review Board (IRB) of each participating institution will continue to review the trial. The TSC will check the consent forms, compliance with the protocol, planned surgical interventions, and quality of data collected in the case report forms at least annually.

#### Composition of the data monitoring committee, its role, and its reporting structure

The Data Safety and Monitoring Board is independent of the sponsor, has not worked with the study team, and does not have competing interests. The Data Safety and Monitoring Board will meet every 6 months to review the study procedures and adverse events (AEs). Each AE is to be classified by the investigator according to the Common Terminology Criteria, version 5.0 [14]. Serious AEs and AEs of special interest are captured and processed until the last visit of the last patient. A final list will be provided to the ethics committee. All serious AEs will be additionally graded according to the Clavien-Dindo classification of postoperative complications.

#### Plans to promote participant retention and complete follow‑up

During follow-up, several strategies, such as collecting detailed contact preferences and sending text messages in advance, will be used to maximise participant retention. Up to five contact attempts will be made before participants are considered lost to follow-up.

### Sample size estimation

This study aimed to test the superiority of the non-muscle-cutting periumbilical transverse incision over the midline incision, and the sample size was estimated based on the primary outcome (the incidence of incisional hernia at 12 months after surgery). Our previous retrospective study showed that the incidence of incisional hernias was 2.4% and 14.9% in non-muscle-cutting periumbilical transverse and midline incisions, respectively [6]. For this study, a conservative estimate rounded these incidences up to 2.5% and 15%, respectively. The sample size calculations were conducted in R using the pwr (pwr.2p.test); the required sample size for a superiority trial was determined to be 158 patients using power analysis (power = 85%, *ɑ* = 0.05, two-sided), and the dropout rate was anticipated at 10%. Finally, a total of 176 patients are required for randomisation.

### Statistical analysis

Data analysis will be performed using primary analysis (ITT), followed by secondary analysis (as-treated principles). The ITT set includes all patients who were randomised regardless of whether they received each incision, and the “per-protocol” analysis set includes patients who were treated according to protocol, excluding major protocol violations. Given our expectation, very few patients will crossover between incision types. All baseline and outcome data will be presented using frequencies with proportions for categorical variables and means with standard deviations for continuous data (or medians with interquartile ranges, whichever is more appropriate). The patients will be compared based on their baseline characteristics, including age, sex, body mass index, American Society of Anaesthesiologists classification, preoperative treatment (none, radiotherapy, or chemotherapy), comorbidities, medications, history of previous abdominal surgery, location of the tumour (right or left), and baseline short form-12 health survey questionnaire to determine the balance between the two groups. The primary outcome (the incidence of incisional hernia) will be analysed using the chi-square test. The secondary outcomes will be analysed using the chi-square test for categorical variables (i.e. type of surgery, resection, and anastomosis, incidence of 30-day postoperative complications, histologic type, and depth of the tumour), and Student’s *t*-test or Mann–Whitney *U* test for quantitative variables (i.e. operative time, length of hospital stay, blood loss, postoperative pain scores, size of the tumour, and the number of lymph nodes) as appropriate. In accordance with symptoms, each incision type will be evaluated during follow-up after surgery. However, it is not expected that there will be missing data relating to the primary outcomes. For other possible missing data, multiple imputations will be made, based on the assumption that the data are missing at random.

Subgroup analyses will be conducted for each randomisation stratum. The results will be evaluated at a significance threshold of *p* < 0.05 (two-sided). All of the statistical analyses will be performed using *R* statistical software, version 3.4.3 (R Foundation for Statistical Computing).

### Patient and public involvement

Patients and the general public were not involved in any part of the planning, conducting, reporting, or disseminating of the results of this study.

## Ethics and dissemination

This trial has been approved by the IRB of Chonnam National University Hwasun Hospital for clinical trials (IRB No. CNUHH 2021–009) on January 19, 2021, and the IRBs of each participating institution. The current study protocol version is 2.2, which was approved on April 21, 2021. The study protocol has been registered in the Clinical Research Information Service (registration number: KCT00006082) as of April 12, 2021. This study will be conducted in accordance with the tenets of the 1964 Declaration of Helsinki and its later amendments. Investigators will provide patients with complete written and oral information on the two types of incision and the process of the trial prior to their participation. The participation of participants in this study will be voluntary, and they can cease at any time without any consequence. We plan to disseminate this information to the relevant patients and healthcare professionals. On request by the participants, we plan to educate them on the trial results during their regular hospital visits at the outpatient clinic. The results will be published in a peer-reviewed journal and presented at pertinent national and international scientific meetings as posters or oral presentations. If a suitable research and data protection strategy is agreed upon, study data may be made available after a request has been made to the principal investigator.

## Discussion

We expect that the non-muscle-cutting periumbilical transverse incision will reduce the incidence of incisional hernias as compared to the midline incision. Previous randomised controlled trials have not clearly demonstrated the effect of a transverse incision in laparoscopic colon cancer surgery on reducing the incidence of incisional hernia. Lee et al. conducted a systematic review including 17 studies and found that the pooled incidence of incisional hernia was 10.6%, 3.7%, and 0.9% for midline, transverse, and Pfannenstiel incisions, respectively [15]. Subsequently, the same authors conducted a prospective randomised controlled study comparing the incidence of incisional hernia between midline and transverse incisions during laparoscopic colon resection surgery [4]. The study reported that the incidence of incisional hernia in the transverse and midline incisions was 2% and 8%, respectively, without statistical significance in the ITT analysis. In the per-protocol analysis, the incidence of incisional hernia in the transverse incision group was significantly lower than that in the midline incision group (2% vs. 15%; *p* = 0.013). However, the cosmesis was significantly worse in the transverse incision group. In previous studies, a muscle-splitting transverse incision was usually created lateral to the linea semilunaris and rectus sheath or in the iliac fossa, which is different from our new incision method. Our incision is created within the rectus muscle. We devised this method because the periumbilical location of the wound is universally feasible for specimen extraction from any location of the colon, as it has been used for single-incision surgery. Moreover, our non-muscle-cutting periumbilical transverse incision method differs from the previous transverse incision for open surgery, in which muscle cutting is performed when crossing the rectus muscle [5, 15]. The bursting pressure during coughing or straining would be dispersed on the sutured fascia layers and rectus muscles by preserving an intact muscle layer. This method, in addition to suturing each rectus sheath separately, might prevent the incidence of an incisional hernia.

The additional use of a CT scan to diagnose incisional hernia has been shown to increase the sensitivity of diagnosing incisional hernia as compared with physical examination alone [16]. This is the reason why this study will enrol patients who undergo surgery for colon cancer and exclude patients with benign disease. These inclusion criteria will enable the regular examination of wounds and the performance of imaging studies. Patients with colon cancer will receive standard regular follow-up after surgical resection of the primary tumour at 3-month intervals for physical examinations and 6-month intervals for CT scans during the first 2 years. We set the time point for the primary outcome of the incidence of incisional hernia as 12 months after surgery. A systematic review found that studies with significantly longer follow-up periods do not report a significantly greater incidence of incisional hernia than those with shorter follow-up periods [15]. Other studies showed that approximately half of the incisional hernias develop during an additional follow-up of 2 years [17, 18]. As we will diagnose incisional hernias using both physical examination and CT scans, the increased diagnostic sensitivity might reduce the interval change since we will check the primary endpoint at 12 months after surgery. Still, we will perform a secondary analysis of the long-term incidence of incisional hernia at 3 years, which will further define the optimal timing to diagnose incisional hernia after surgery.

Patients with rectal cancer are excluded from this study. Rectal cancer has various clinical factors during treatment, such as perioperative treatment, which sometimes requires different schedules of follow-up for colon cancer. Moreover, surgery for rectal cancer has the potential for faecal diversion during surgery or when accounting for postoperative anastomotic leakage and has a higher risk of abdominal infectious complications (i.e. pelvic abscess) than colon cancer [19]. The site for protective faecal diversion is usually outside the midline and can be used for specimen extraction to avoid two small incisions. Nonetheless, the midline incision is frequently used for rectal cancer surgery [20]. If this study proves the superiority of the non-muscle-cutting periumbilical transverse incision, it can be used for rectal cancer surgery instead of the midline incision, preserving its benefits for patients with rectal cancer.

This study has several limitations. The sample size calculation for this study was based on the data from our retrospective study. The wound closure procedures used in the previous study were not standardised and were performed by heterogeneous surgeons with various levels of experience. Therefore, the sample size may have been underestimated or overestimated. Another limitation is that the patients and surgical team will not be blinded. Blinding the surgical team is impossible because they have to perform the procedure. However, the surgery team will be separated from the outcome assessors of CT scans, who will be blinded to the group allocation, and the data analysis will be performed by a separate statistical analysis team.

In this study, we aimed to confirm the superiority of the non-muscle-cutting periumbilical transverse incision over the midline incision concerning the incidence of incisional hernias (both symptomatic and radiologic hernias). We expect that our findings will help surgeons determine the optimal location of small incisions for minimally invasive colon cancer surgery.

## Trial status

This trial is currently open for recruitment. We anticipate reaching our maximum number of included patients by December 2023.

## Data Availability

Any data required to support the protocol can be provided upon request. The datasets analysed during the current study and the statistical codes are available from the corresponding author on reasonable request, as is the full protocol.

## Abbreviations

AE: Adverse event
CT: Computed tomography
ITT: Intention-to-treat
